# Cambrian carbonaceous protoconodonts and the early fossil record of the Chaetognatha

**DOI:** 10.1098/rspb.2024.2386

**Published:** 2025-02-19

**Authors:** Ben J. Slater

**Affiliations:** ^1^ Department of Earth Sciences, Palaeobiology, Uppsala University, Uppsala, Sweden

**Keywords:** Cambrian, Ediacaran, Chaetognatha, protoconodont, Gnathifera, Bilateria, small carbonaceous fossils

## Abstract

Fossil remains from the early Cambrian Period suggest an ancient origin for the phylum Chaetognatha. As macrofossils, Cambrian chaetognaths are restricted to just a few Konservat-Lagerstätten sites, yet the dispersed grasping spines characteristic of this clade are relatively common as phosphatized ‘protoconodonts’. Here, an abundance of protoconodonts are described, but preserved in an entirely different manner, as ‘small carbonaceous fossils’ (SCFs) extracted from Cambrian Fortunian and Stage 4 mudrocks of Estonia and Sweden, respectively. Preservation among small carbonaceous fossils is substantial, representing an alternative but overlooked resource for tracing the origins of the chaetognath clade. Importantly, small carbonaceous fossils are abundant in normal marine siliciclastic deposits, in which conventionally studied phosphatic protoconodont fossils are scarce. Recent advances in constraining the phylogenetic position of chaetognaths suggest a relationship to the clade Gnathifera (gnathostomulids, micrognathozoans, rotifers). Newly emerging small carbonaceous fossil records, therefore, offer the chance to establish important calibration points for the divergence of deep bilaterian clades, including Protostomia, Lophotrochozoa and Gnathifera. Protoconodonts are especially valuable in this respect given their appearance close to, or prior to, the Ediacaran–Cambrian boundary. A first compilation of small carbonaceous fossil protoconodont records suggests chaetognath-like bilaterians had evolved by the latest Ediacaran (approximately 555–545 Ma), while the jaw complex possessed by the chaetognath crown-group emerged at least 520 Ma.

## 1. Introduction

Protoconodonts are among the oldest recognizably bilaterian remains in the fossil record [1–3]. Their appearance as abundant phosphatic/apatitic spines in basal Cambrian strata has become increasingly important in determining the stratigraphy of the Cambrian boundary [4]. The term ‘protoconodont’ was first introduced by Bengtson [5] to delineate an assortment of morphologically simple ‘conodont’ elements that appeared to grow via basal–internal accretion of lamellae. At this stage, the term carried an implicitly phylogenetic connotation, and protoconodonts were hypothesized to be ancestral to later paraconodonts and euconodonts [5]. Following detailed histological investigations by Szaniawski [6–9], alongside the discovery of various clustered arrays ([10]; fig. 2I–M of [9]), it became increasingly clear that most protoconodonts were actually sourced from the grasping spines of chaetognath arrow-worms [9,11,12]. Given the protostome affinity of chaetognaths [13], it is, therefore, now generally accepted that protoconodonts are only distantly related to either euconodonts or paraconodonts, which instead reside among the chordates [14].

Until recently, there was little consensus on precisely how chaetognaths relate to other bilaterian clades [15]. Traditionally, chaetognaths had been placed with the deuterostomes based on their early embryological development, but with the advent of molecular sequence data, the hypothesized position of chaetognaths has shifted. Early studies suggested a divergence prior to the evolution of coelomate metazoans [16], then later as sister to the protostomes [17], and more recently chaetognaths have been placed within the protostomes [18]. Further advances in constraining their phylogenetic position using transcriptomes from multiple chaetognath taxa have resolved the Chaetognatha as a clade closely allied to the Gnathifera—a lophotrochozoan clade comprising rotifers, gnathostomulids and micrognathozoans—with chaetognaths either residing within the Gnathifera [13] or as sister to the Gnathifera [19,20].

**Figure 1.**
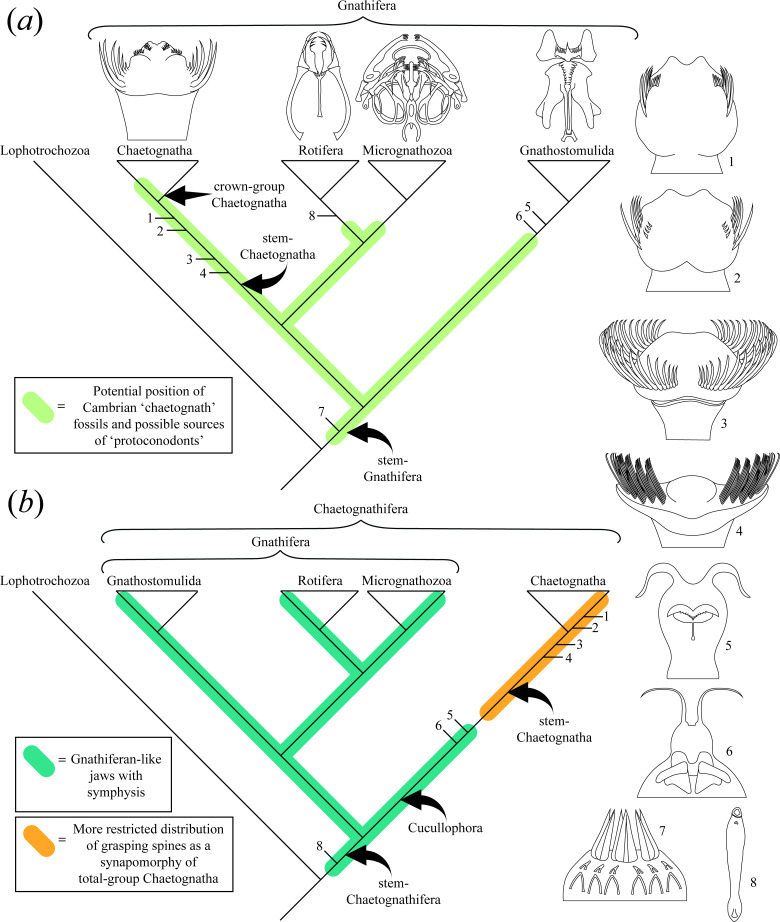
Alternative schematic cladograms highlighting potential positions of Cambrian fossil chaetognath-like taxa. Note that Cambrian ‘protoconodonts’ could, in principle, derive from a broad range of stem-gnathiferans depending on the plesiomorphy of such spines. 1, *Protosagitta spinosa*; 2, *Eognathacantha ercainella*; 3, *Capinatator praetermissus*; 4, *Ankalodous sericus*; 5, *Amiskwia sagittiformis*; 6, *Timorebestia koprii*; 7, *Dakorhachis thambus* and 8, *Inquicus fellatus*. (*a*) Gnathifera hypothesis based on phylogeny in fig. 1 of [13]. Potential affinities of Cambrian fossil ‘chaetognaths’ are depicted as upper stem-chaetognaths (*Protosagitta* and *Eognathacantha*), stem-chaetognaths (*Capinatator* and *Ankalodus*), stem-gnathostomulids (*Amiskwia*) and stem-gnathiferans (*Dakorhachis*). The parasitic taxon *Inquicus* may be secondarily simplified. (*b*) Chaetognathifera hypothesis, based on parsimony analysis in fig. 1 of [20]. In [20], Cambrian fossil ‘chaetognath’ taxa were found to represent crown-group chaetognaths (*Protosagitta*), total-group chaetognaths (*Capinatator* and *Ankalodus*), sister taxa to the Chaetognatha (*Amiskwia*) and sister taxa to the Chaetognatha + Gnathifera (*Inquicus*). See also [21] which argues that *Amiskwia* and *Timorebestia* are stem-chaetognaths, implying a more restricted source of fossil grasping spines. Line drawings for Chaetognatha, Rotifera, Micrognathozoa and Gnathustomulida after various sources. Fossil line drawings: 1 (after material in [12,22,23]), 2 (after material in [24]), 3 (based on reconstruction in [25]), 4 (based on reconstruction in [23]), 5 (after fig. 4 of [20]), 6 (after fig. 4 of [21]), 7 (based on reconstruction in [26]) and 8 (after material in [27]).

In light of chaetognath affinities, a reasonable question is whether the complex ‘jaws’ of chaetognaths, rotifers, gnathostomulids and micrognathozoans evolved from a homologous structure that was present within Cambrian (or late Ediacaran) ancestors of chaetognaths and gnathiferans (figure 1). This raises the further question of whether Cambrian protoconodonts (or indeed other early fossil ‘chaetognaths’) are sourced from stem-chaetognaths, crown-chaetognaths, stem-members of various gnathiferan clades or stem-gnathiferans. A number of partially and completely articulated Cambrian fossil chaetognaths have now been described from Konservat-Lagerstätten [21–26,28]. Among these Cambrian forms there is substantial variation in the organization of the feeding apparatus. For instance, *Capinatator praetermissus* from the Burgess Shale possessed paired bundles of grasping spines with up to ~50 spines borne by a single individual [25]. Similarly, *Ankalodous sericus* from the Chengjiang Lagerstätte displays a comparably large number of bundled grasping spines [23]. Both these Cambrian taxa possessed almost double the number of grasping spines of any known extant chaetognath, yet their overall morphology and suite of characters is suggestive that they were total-group chaetognaths. Contemporaneous with these forms are taxa such as *Protosagitta spinosa* [12,22,23] and *Eognathacantha ercainella* [24] which appear to already possess the classical feeding apparatus of crown-chaetognaths, comprising differentiated ‘teeth’ and a smaller number of grasping spines. Other broadly chaetognath-like Cambrian forms, such as the famous Burgess Shale taxon *Amiskwia sagittiformis* [29] and recently described *Timorebestia koprii* [21] appear to possess somewhat gnathustomulid-like jaws (fig. 5 of [30,31]), and lack the characteristic grasping spines of crown-chaetognaths. Recent analysis by Bekkouche & Gąsiorowski [20] and Park *et al*. [21] nevertheless resolve *Amiskwia* and *Timorebestia* as stem-chaetognaths, with chaetognaths + *Amiskwia*/*Timorebestia* as sister to the Gnathifera. Bekkouche & Gąsiorowski [20] have also argued, based on their differing microstructure, that the jaws of gnathiferans and chaetognaths are not homologous. This implies that in the stem leading to chaetognaths, early total-group chaetognaths had gnathiferan-like internal jaws capable of symphysis (*Amiskwia* and *Timorebestia*), which were later lost, with crownward chaetognaths (e.g. *Capinatator*) subsequently acquiring a grasping apparatus (figure 1). This would mean that even the earliest fossil protoconodonts were sourced from relatively crownward stem-chaetognaths. Alternatively, if chaetognaths reside within the Gnathifera (cf. [13]), then grasping spines (protoconodonts) may have a more plesiomorphic distribution (figure 1) and were later lost among the gnathifera (and perhaps in a plesion leading to *Amiskwia* and *Timorebestia*).

Clearly, the Cambrian fossil record is crucial to disentangling the precise details of early chaetognath and gnathiferan evolution (figure 1). Nevertheless, since articulated macrofossils of Cambrian ‘chaetognaths’ are restricted to a handful of Konservat-Lagerstätten, expanding our knowledge of Cambrian protoconodonts is essential to resolving the temporal dimension of this radiation. A deeper understanding of the Cambrian protoconodont record offers the chance to establish important calibration points for the divergence of deep bilaterian clades, including crown-group Bilateria, Protostomia and Lophotrochozoa, as well as total-group Gnathifera and Chaetognatha. Protoconodonts are especially valuable in this respect given their early appearance close to, or prior to, the Ediacaran–Cambrian boundary [3].

Most protoconodonts are extracted (using weak acetic acid) as phosphatic or phosphatized small shelly fossils (SSFs) [32–38]. Among these disarticulated SSFs, grasping spine morphologies exhibit a substantially greater disparity than is seen among *in situ* articulated macrofossils, hinting at a rich cryptic diversity of chaetognath-like forms in Cambrian oceans. There is mounting evidence that many SSF protoconodonts were originally organic and are only secondarily phosphatized [39,40]. Consequently, preservation of SSF protoconodonts is likely to be controlled by the prevalence of phosphatic deposits [41–43]. Yet SSFs are not the only means of accessing this hidden diversity. A somewhat under-utilized source of information regarding Cambrian chaetognaths is the dispersed organically preserved protoconodonts found among the emerging record of small carbonaceous fossils (SCFs) [44].

Although often fragmentary, SCFs can preserve detailed microanatomical structures—in some cases exceeding the level of detail seen in Burgess Shale-type deposits. Furthermore, because of their small size and potential for biostratinomic dispersal, SCFs are found in a broad spectrum of marine shales and mudrocks. Consequently, SCFs are crucial for detecting non-mineralized aspects of diversity outside rare and spatio-temporally restricted Konservat-Lagerstätten sites. For instance, exploration of this emerging SCF record has uncovered a variety of organically preserved animal mouthparts and feeding structures [45–49] that are otherwise unknown from the wider fossil record.

Here, I describe protoconodonts preserved as SCFs from Cambrian strata of Estonia and Sweden, which illustrate the key features of protoconodonts preserved in this fashion. Furthermore, scattered reports of carbonaceous protoconodonts among the broader Ediacaran–Cambrian archives of organic microfossils are collated here for the first time to establish a detailed temporal accounting. The significance of early protoconodonts to constraining the timing of the evolution of chaetognaths, gnathiferans and the Bilateria is discussed in detail.

## 2. Material and methods

**Figure 2.**
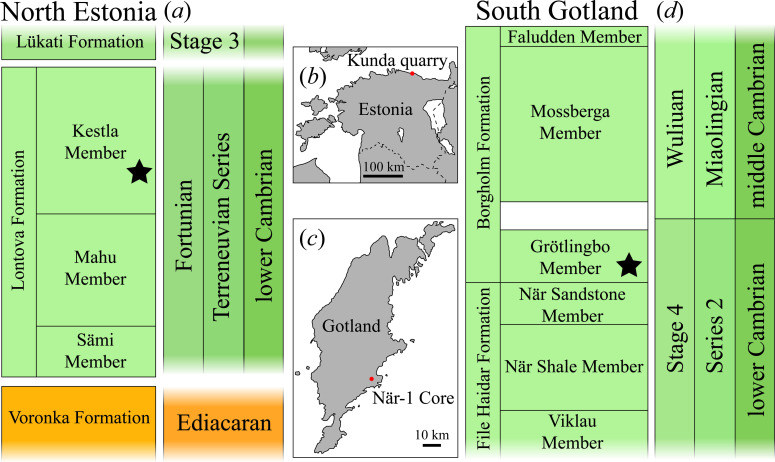
(*a*) Stratigraphy of the Ediacaran–Cambrian sedimentary succession in northern Estonia. (*b*) Map of Estonia indicating the position of Kunda quarry. (*c*) Map of Gotland (Sweden) indicating the position of När-1 drillcore. (*d*) Stratigraphy of the Cambrian sedimentary succession in southern Gotland (after fig. 12 of [50]). Black stars denote members sampled for small carbonaceous fossils (SCFs).

Cambrian Fortunian protoconodonts were hydrofluoric acid-extracted from the ‘blue clays’ of the Kestla Member of the Lontova Formation, from the Kunda quarry in northern Estonia (figure 2). The Lontova Formation comprises poorly lithified bluish-grey laminated clay-rich strata, subdivided into three members (differing slightly in lithology and fossil contents) including an upper Kestla Member, a middle Mahu Member and lowermost Sämi Member [51], and represents deposition in a shallow epicontinental sea. Acritarchs from the Lontova Formation include typical Fortunian-age taxa *Granomarginata prima* and *Asteridium tornatum* [51]. Macrofossils are dominated by the agglutinated tube *Platysolenites*, and the carbonaceous tube *Sabellidites*, a marker for the lowermost Cambrian, is common in the basal Sämi Member. The Lontova Formation overlies the Ediacaran age Voronka Formation, and is capped by the Cambrian Stage 3 Lükati Formation (figure 2).

Younger protoconodonts were sourced from the lowermost Borgholm Formation on the island of Gotland, where sediments represent deposition in a shallow epicontinental sea. On Gotland, Cambrian strata are not exposed at the surface, and are accessible only from subsurface cores [52,53]. The Cambrian succession on Gotland varies in thickness between 140 and 225 m [54], resting unconformably on a Proterozoic basement, and is capped by Ordovician or Silurian sediments. In 1968, the borehole Närborrningen-1 (När-1) was drilled in southeastern Gotland (figure 2; [54]) intersecting 175.15 m of Cambrian rocks, consisting of 95 m of the lower Cambrian File Haidar Formation, 78 m of lower/middle Cambrian Borgholm Formation, and 2 m of upper Cambrian Alum Shale and grey limestone from the *Agnostus pisiformis* Zone [54,55]. Over most of its extent, the Borgholm Formation is separated from the underlying File Haidar Formation by a regional unconformity known as the ‘Hawke Bay event’ considered to mark the Cambrian Stage 4–Wuliuan boundary [50]. The lowermost Grötlingbo Member of the Borgholm Formation nevertheless underlies the Hawke Bay unconformity, and has previously been considered the upper part of the File Haidar Formation [54,56,57]—indeed the Grötlingbo Member lies within the *Comluella-Ellipsocephalus lunatus* Trilobite Zone corresponding to Cambrian Stage 4 [55]. On lithological grounds, however, the Grötlingbo Member has been subsumed within the otherwise Wuliuan-age Borgholm Formation [55]. SCF protoconodonts recovered here are from the basal part of the Borgholm Formation, considered to represent the latest part of early Cambrian deposition in this region.

A sample from the Kunda quarry (0.9 m depth from the top), and a sample from 537.28 m depth in the När-1 drillcore were each macerated in 40% concentrated hydrofluoric acid (50 g per sample). The resulting organic slurry was neutralized and sieved using a 50 µm mesh. Both samples produced abundant carbonaceous spines, alongside other organic microfossils. SCFs were picked and permanently mounted on glass slides following the procedure outlined in [45]. Microfossils were studied and photographed using a Leica-DM-2500 microscope. Slides are stored in the palaeontological collections at the Museum of Evolution (PMU), Uppsala University, Sweden (electronic supplementary material, S1).

## 3. Small carbonaceous fossils

### (a) Carbonaceous protoconodonts (Cambrian Fortunian)

**Figure 3.**
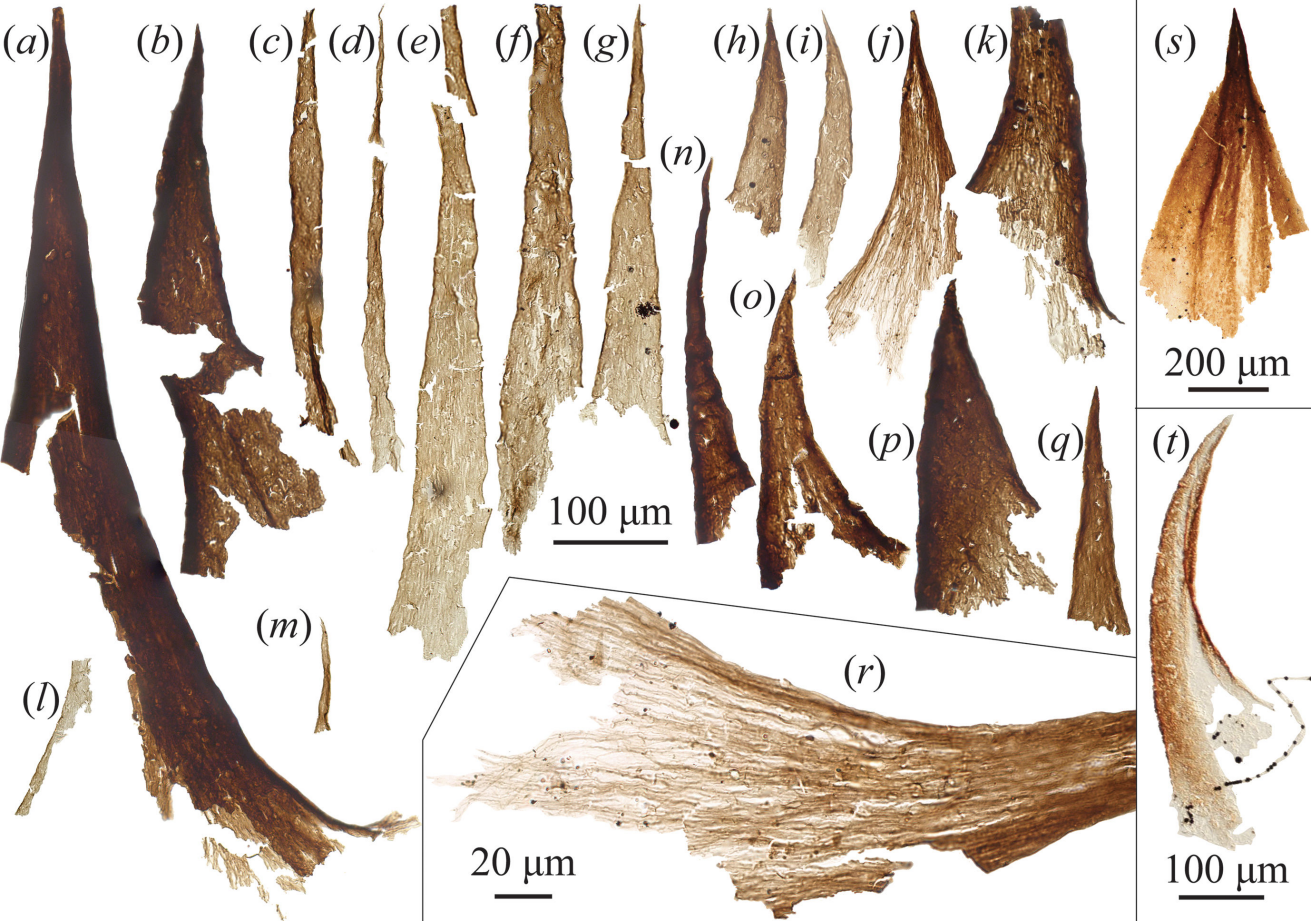
(*a–r*) Organically preserved protoconodonts from the Cambrian Fortunian age Kestla Member of the Lontova Formation, Estonia, and (*s,t*) comparable Ediacaran SCFs. (*r*) Close-up of fibrous microstructure in (*j*). (*s*) Comparable protoconodont-like bilaterian sclerite from the late Ediacaran (Redkino-age) of Finland (distal portion particularly reminiscent of protoconodonts). (*t*) Protoconodont-like spine from the late Ediacaran Staraya Russa Formation (Redkino-age) of Leningrad Oblast, Russia, reproduced with permission, Golubkova *et al*. [58] (specimen numbers in electronic supplementary material, S1). Scale bars = 100 µm for (*a–q*,*t*), 20 µm for (*r*), 200 µm for (*s*).

The sample from the Lontova Formation produced abundant flattened organic spines (widths approximately 20−160 μm, lengths approximately 100−830 μm), many exhibiting a slightly curved horn-shaped outline (figure 3*a*–*r*). At least two layers are evident in the spines, a circumferential optically denser outer layer, and a lighter inner layer (figure 3*a*,*j*,*k*,*r*). When viewed in higher magnification (figure 3*r*), the microstructure of these layers is revealed to comprise thin, densely packed longitudinally arranged fibres. These spines are identical to those previously detected from the Lontova Formation and Fortunian SCF assemblages elsewhere (e.g. [44,59]) that have been assigned to the protoconodont taxon *Protohertzina compressa* [44]. Such spines are also somewhat comparable to those detected in underlying Ediacaran strata (e.g. figure 3*s*,*t*) [3]. These oldest spines have been detected in Kotlin-age formations dated to 551−548 Ma using uranium-lead zircon ages from tuffs in equivalent units (e.g. in Ukraine [60]), as well as older Redkino-age formations that correlate to 555.3 Ma dated tuffs in equivalent Ediacaran units (e.g. the White Sea region in northwest Russia [61]).

### (b) Carbonaceous protoconodonts (Cambrian Stage 4)

**Figure 4.**
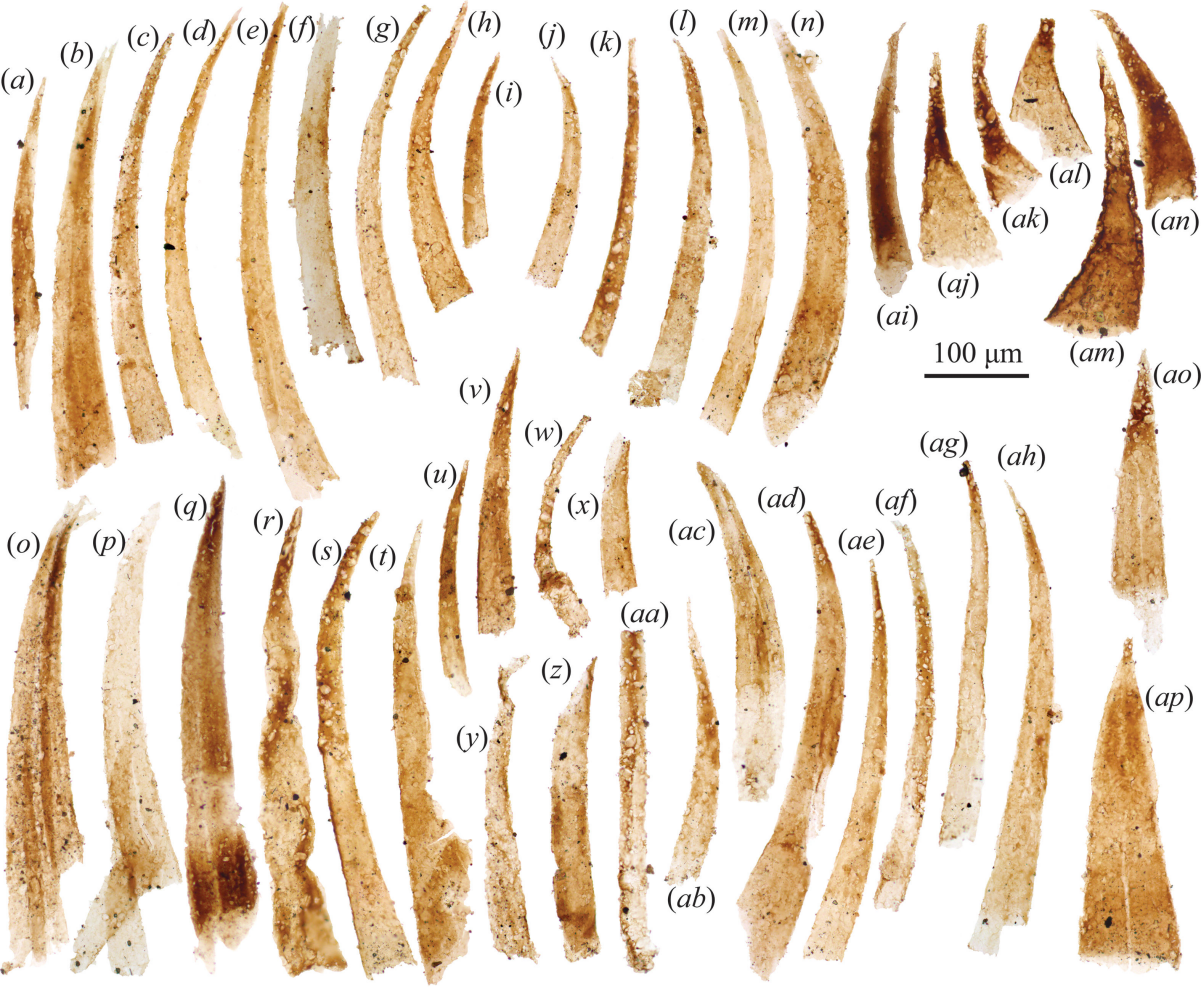
(*a–ah*) Organically preserved protoconodonts from the Cambrian Stage 4 Grötlingbo Member of the Borgholm Formation, Gotland, Sweden. (*ai–ap*) Co-occurring organic spines which may relate to protoconodonts (see §4; specimen numbers in electronic supplementary material, S1). Scale bar = 100 µm.

Thin, gently curving elongate spines (widths approximately 17−120 μm, lengths approximately 100−830 μm) were abundant in the Borgholm Formation sample (figures 4*a–an*, 5). A subset of these spines is found as articulated pairs and clusters (figures 4*b*,*o*,*p,ac* and 5*a,j,h*), with up to four discernible spines stacked into a cluster. Stouter spines with a greater width–length ratio tend to be darker and more heavily sclerotized (figure 4*ai–an*). Where the basal portion of spines is intact (e.g. figure 4*a*,*n*), it appears as a teardrop-shaped outline. Many spines have a pock-marked appearance (e.g. figure 4*k*,*s*), presumably the result of adhering pyrite crystals dislodged or removed during acid digestion.

**Figure 5.**
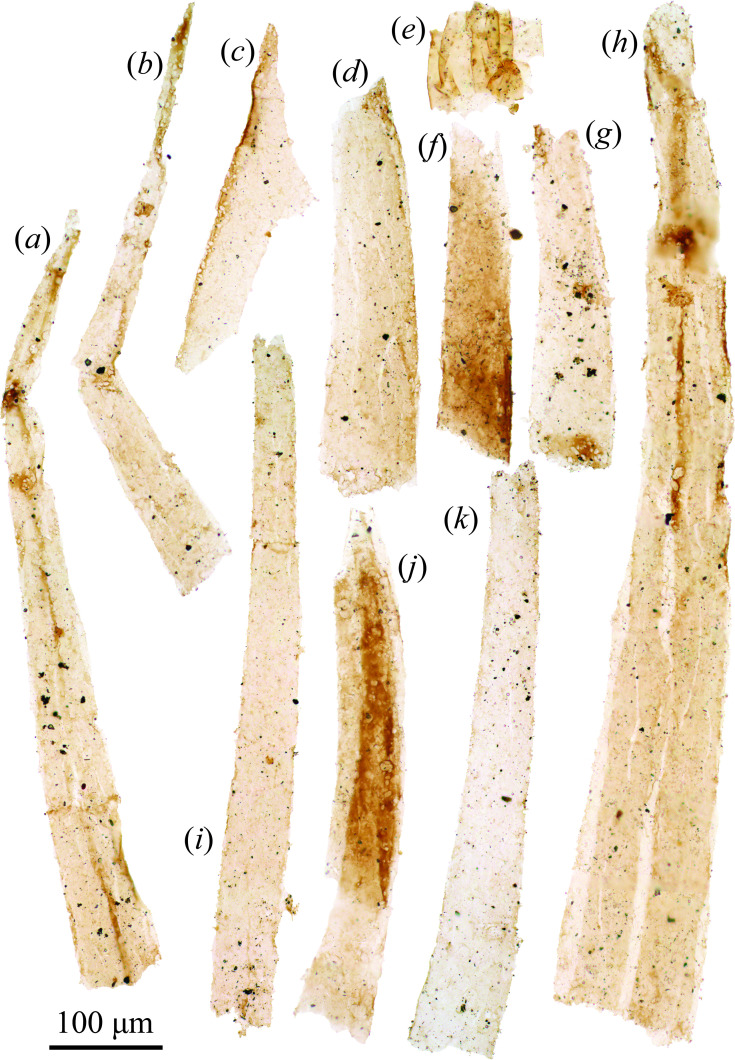
(*a–k*) Relatively large SCF protoconodonts from the Cambrian Stage 4 Grötlingbo Member of the Borgholm Formation, Gotland, Sweden (specimen numbers in electronic supplementary material, S1). Scale bar = 100 µm.

Spines of this morphology have also been recovered from the File Haidar–Borgholm Formation boundary from Östergotland (see fig. 9*a*–*t* of [53]), and were compared with protoconodonts. The slender spines recovered here are close in overall morphology to the protoconodont *Phakelodus* [62] found as isolated spines and semi-articulated clusters (figs. 3 and 5 of [63]; figs. 2*i–k* and 5*e* of [9]), as well as the *in situ* grasping spines of *Ankalodous sericus*, a multi-jawed chaetognath from the Chengjiang Biota [23] (compare also to an articulated chaetognath grasping apparatus from the Burgess Shale in fig. 1*c* of [28]). One specimen (figure 4*r*) potentially exhibits a spirally twisted habit, as seen among similar organic spines assigned to *Ceratophyton* sp. ‘type-B’ from the Random Formation in Newfoundland (fig. 7*a* of [64]).

**Figure 6.**
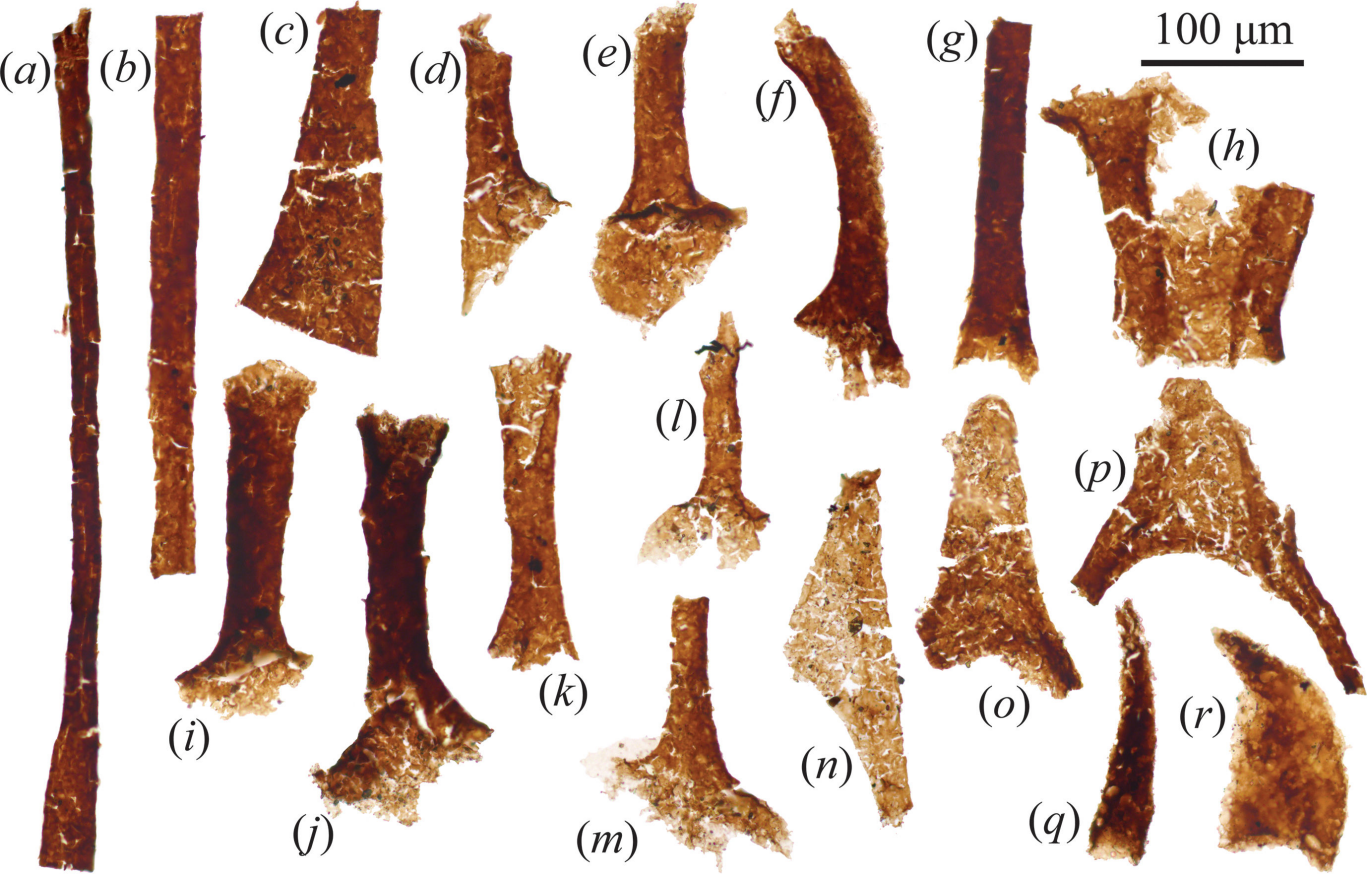
(*a–r*) Rod-shaped elements co-occurring with SCF protoconodonts from the Cambrian Stage 4 Grötlingbo Member of the Borgholm Formation, Gotland, Sweden (specimen numbers in electronic supplementary material, S1). Scale bar = 100 µm.

Two broadly triangular elements (figure 4*ao,ap*) are distinguished by their lack of curvature and longitudinally developed midline. Comparable SCFs were described by Slater *et al*. [65] as the dorsoventrally flattened cardinal spines of *Isoxys*-like bivalved arthropods from the early Cambrian Buen Formation of North Greenland (see fig. 3*w*–*ak* of [65]; fig. 6*v*–*af* of [47]). Under this interpretation, the midline would represent the carapace hinge-line (e.g. fig. 4*c* of [66]). However, it is possible that these Borgholm spines (figure 4*ao,ap*) reflect protoconodonts flattened at an angle that simply removed any curvature. Comparably shaped protoconodont-like spines with a triangular outline and prominent midline (jugal line?) are even found in the articulated feeding apparatus of *Dakorhachis thambus* from the Guzhangian Weeks Formation of Utah (fig. 4 of [26]).

### (c) Co-occurring elements (Cambrian Stage 4)

If chaetognaths are gnathiferans [13], then chaetognath jaws could at some level be homologous with the cuticular jaws of rotifers, gnathostomulids and micrognathozoans [67,68]. In this view, the complex feeding apparatus of crown-chaetognaths was likely partly assembled within stem-gnathifera (or stem-Chaetognathifera cf. [20]), as well as stem-Chaetognatha (figure 1). Cambrian fossil ‘stem-chaetognaths’ may therefore exhibit character combinations that do not conform to the expectations of crown-group or extant chaetognath jaws. Curiously, the fossil grasping spines recovered here from the Borgholm Formation co-occur with rod-like and V-shaped SCF elements that exhibit broad similarities with structures found in the feeding apparatus of both extant and extinct gnathiferan taxa (figure 6). These rod-shaped elements (140–530 µm length, 10−90 µm width) exhibit flared termini (figure 6), alongside examples displaying bifurcation, with adjoined rods forming V-shaped or curved connections (figure 6*h*,*n*–*p*).

The trophi of extant rotifers contains comparable rod-shaped, V-shaped and curved elements such as the fulcrum, manubrium and ramal alula (e.g. fig. 4.39 of [69–71]). Similarly, the feeding structures of gnathustomulids [72,73] and micrognathozoans (e.g. [74]; fig. 8*a* of [75]) also bear an array of branching and linear rod-shaped extensions. A similar co-occurrence of carbonaceous protoconodonts with rod-shaped and V-shaped structures was reported from the File Haidar/Borgholm Formation boundary elsewhere in Sweden, from Östergotland (fig. 9A–T of [53]). Based on such disarticulated material, it is not currently possible to determine whether these elements genuinely comprise parts of an extended jaw apparatus (though repeated co-occurrence with carbonaceous protoconodonts is suggestive of an association). It is important nevertheless, to consider whether such co-occurring material could derive from a broader jaw apparatus absent among extant chaetognaths. Interestingly, the articulated feeding apparatus of the Cambrian ‘gnathiferan-chaetognath’ *Dakorhachis thambus* also exhibits protoconodont-like spines alongside rod-shaped and V-shaped elements (fig. 4 of [26]). If considered together, the SCFs described here could conceivably represent a feeding apparatus akin to some of the ‘hypothetical-intermediary’ stages proposed by Conway Morris *et al*. [26] as transitions from ancestral Gnathifera to chaetognath-like and gnathiferan-like jaws (fig. 8 of [26]). Pending the discovery of fully articulated body fossils, identifying repeated co-associations of elements found with Cambrian phosphatic and organic protoconodonts may eventually reveal novel feeding apparatus morphologies from early in chaetognath evolution (a situation analogous to the case of disarticulated euconodonts until bedding-plane articulated apparatus were discovered [76–78], and later complete body fossils [79,80]).

## 4. Discussion

### (a) Microstructure of carbonaceous protoconodonts

One advantage of SCF preservation over phosphatic SSFs is that it enables the study of internal microstructures using transmitted light microscopy. When imaged in high magnification, it is clear that carbonaceous protoconodonts possess a denser outer layer, and at least one lighter inner layer (figure 3). Both these layers appear to be composed of thin, mostly longitudinally arranged fibres, especially visible in the lighter internal layer (figure 3*r*). These layers have frequently become fused together during flattening and diagenesis, but in many specimens, the outer layer is partially missing revealing the more obviously fibrous internal layer (figure 3*r*). Certain specimens possibly represent wholly detached inner layers, where the outer layer has been abraded (figure 3*d*–*g,i,j,r*), affording a detailed view of the fibrous construction. Close examination reveals the fibrils are arranged in longitudinal strips, which are parallel toward the apical portion of the spine, but begin to twist (rotate around the axis of the spine) and become splayed toward the base. In SCF protoconodonts, the spine can appear split lengthways (fig. 3BO–BS of [44]), potentially representing where the outer and inner fibrous layers merge in modern chaetognath grasping spines [9]. The underlying fibrous construction is especially evident in the older Fortunian specimens recovered here (as well as from [44,59]) in contrast to the younger Borgholm Formation specimens. In part, this is likely down to differential taphonomic histories—orange-coloured organic microfossils from the Borgholm Formation tend to be slightly kerogenized, forming a homogeneous layer, whereas the especially low burial temperatures (<50°C) and clay matrix of the Estonian material have conserved finer-scale details.

Notably, several other types of SCF are also known to be constructed from densely packed parallel longitudinal internal channels, including the dorsal sclerites of *Wiwaxia* (fig. 3 of [81]), and various fossilized annelid-like chaetae (fig. 8 of [53]; figs. 8–10 of [81]). This fabric is characteristic of the chaetae (or homologous structures) among a wide range of extant lophotrochozoan taxa [82]. Such chaetae are produced by microvillar secretion within a specialized pocket of cells known as a chaetoblast which adds material at the base, imparting longitudinal internal channels where the microvilli projected into the structure. Intriguingly, examination of the basal region of extant chaetognath grasping spines has shown there are similar microvillar projections emanating from cells surrounding the base of the grasping spine that likewise penetrate into the spine, and presumably secrete the fibrous material [83]. Similarities between the microstructures of the oldest known SCF protoconodonts and that of SCFs derived from lophotrochozoan chaetae could point to a deeper homology between various cuticular structures possessed by chaetognaths, gnathiferans and the Lophotrochozoa (e.g. annelid chaetae, brachiopod setae, chaetal structures in various molluscs, phoronids and bryozoans). Despite this resemblance, the presence of a pulp cavity clearly distinguishes protoconodonts from solid lophotrochozoan chaetae. Moreover, the longitudinal fibres comprising the outer layers of protoconodonts (figure 3*r*) (also seen in the pulp of grasping spines; fig. 3*c,d* [83]) appear to be positive structures rather than the negative longitudinal channels imparted by secretory microvilli in lophotrochozoans.

### (b) Organic preservation of protoconodonts

Most known protoconodont fossils are grasping spines with a phosphatic composition. Recovery of entirely organic protoconodonts here adds further plausibility to claims that phosphate mineralization in protoconodonts is secondary, and therefore diagenetic [39,44]. Indeed, extant chaetognath grasping spines are not mineralized, and comprise outer layers of chitinous material (containing zinc-rich granules) surrounding a central cavity filled with a pulp of the aforementioned fibrous microtubules [83]. Nevertheless, numerous biomineralized invertebrate structures are known to preserve as de-mineralized SCFs (e.g. [84–87]), and experiments have shown that intact organic scaffolds or ‘conchixes’ (resembling SCFs) can be isolated from biomineralized shell material of modern taxa [88]. It is plausible, therefore, that some early chaetognaths may have possessed biophosphatic jaws that still produced SCFs when demineralized during diagenesis.

### (c) Emerging significance of early protoconodont fossils

**Figure 7.**
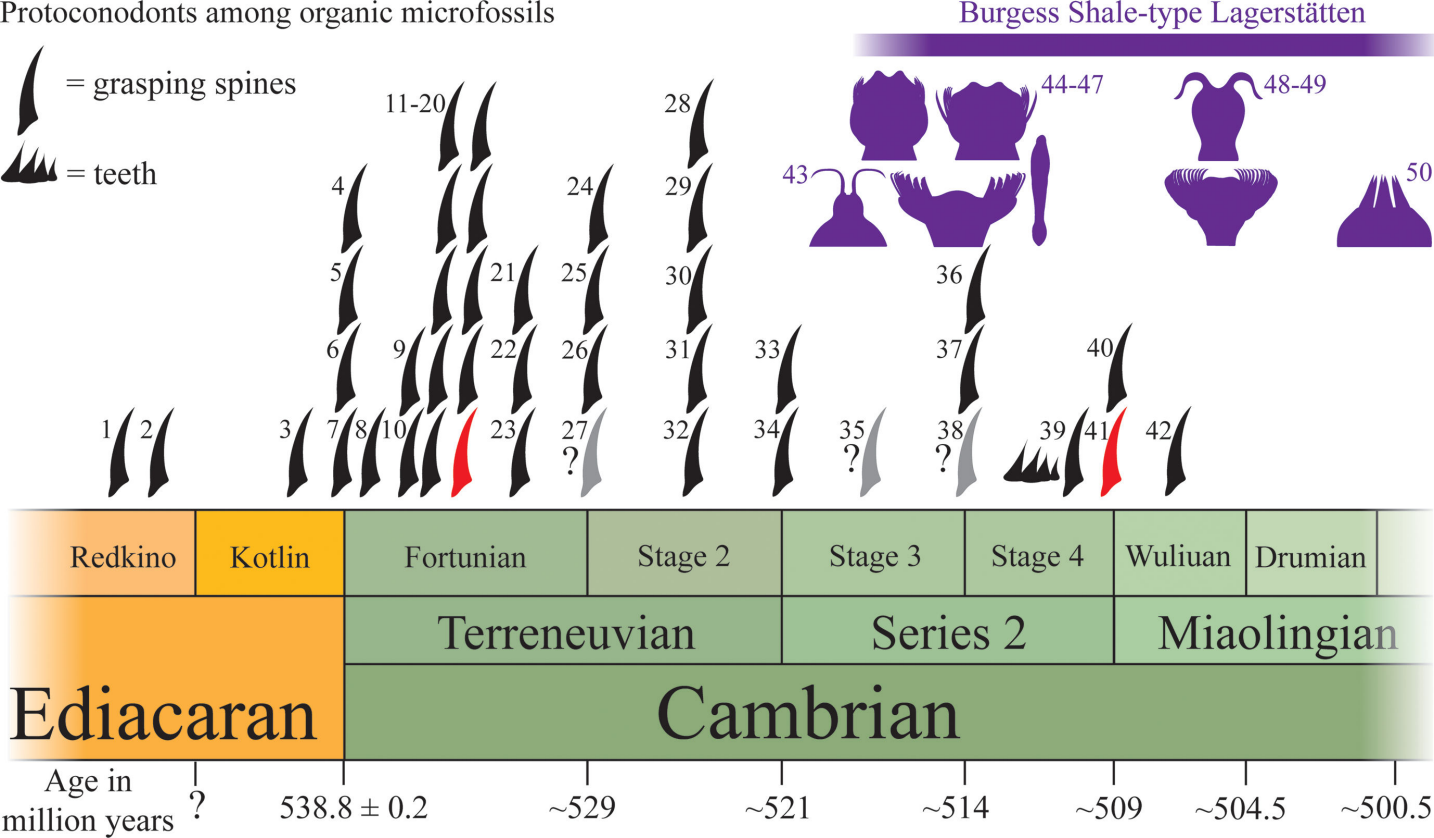
Earliest fossil record of protoconodonts among acid-extracted SCFs. Data collated from both palynological studies and SCF-based investigations throughout the late Ediacaran–Cambrian Miaolingian stratigraphic interval. 1 [89], 2 Staraya Russa Formation [58], 3 [90], 4 [91], 5 [92], 6 [93], 7 [64], 8 [94], 9 [95], Berkuta Formation, 10 [96], 11 [97], 12 [98], 13 [99], 14 [100], 15 [101], 16 [94], 17 [102], 18 [44], Lontova Formation, 19 [44], Voosi Formation, 20, this study, Lontova Formation, 21 [103], 22 [59], 23 [104], 24 [98], Stradech Formation, 25 [105], 26 [106], 27 [107], possible grasping spine but unfigured (*Ceratophyton vernicosum*), 28 [108], 29 [109], 30 [110], 31 [102], 32 [111], 33 [95], 34 [112], 35 [107], possible grasping spine but unfigured, 36 [95], 37 [65], 38 [107], possible grasping spine but unfigured, 39, possible ‘teeth’ and grasping spine [113], 40 [53], 41, this study, Borgholm Formation, 42 [114], 43, Sirius Passet macrofossils, 44−47, Chengjiang macrofossils, 48−49, Burgess Shale macrofossils, 50, Weeks Formation macrofossils. Note some reports document just a few grasping spines or single occurrences (e.g. [99,114]), while others represent detection of numerous grasping spines (e.g. [44], this study). Phosphatized protoconodonts first appear near the base of the Cambrian Fortunian Stage [4].

The first recognizably bilaterian remains in the entire fossil record comprise carbonaceous protoconodont-like spines (figure 7) [3,58,89], and various carbonaceous hooks likely sourced from scalidophoran- or cycloneuralian-like ecdysozoan worms [3]. Outside body fossil remains, characteristic trace fossils also suggest an early (late Ediacaran) emergence of ecdysozoan worms [115–117]. Given the current resolution of chronostratigraphic correlation around this time window, it is often unclear which of these bilaterian ‘spines’—protoconodonts or ecdysozoan scalids—appear first within the fossil record. Ediacaran Redkino-age protoconodont-like spines, however (figure 7), seem to predate the oldest ecdysozoan scalids and traces. In fossil material though, it can often be difficult to distinguish simple hook-shaped ecdysozoan scalids from protoconodonts. Close inspection of protoconodont SCFs reveals that unlike solid-walled ecdysozoan sclerites, they are composed of a multi-layered mass of fibres. Although the outline can be more-or-less hook-shaped in both these SCF types, observations reveal that protoconodont SCFs preserve as compressed cylindrical structures, where depending upon the angle of flattening an ovoid basal cross-section is evident in some specimens (figure 4*n,af*). In contrast, priapulid-like scalids (which grow by moulting) appear as a concave arch with a flattened basal spur that originally connected to the body cuticle in life [118].

Ediacaran age SCFs include several protoconodont-like forms, the oldest of which are found within strata belonging to the local Baltic ‘Redkino’ stage and overlying ‘Kotlin’ stage of the late Ediacaran (figure 3*s*,*t*; [58,89,90]), alongside occurrences close to the Ediacaran–Cambrian boundary [64,91–93]. At present, none of these are known from articulated arrays, and so the broader morphology of the producer organism is entirely unknown. However, the fibrous construction of these microfossils—which resembles the layers of chaetognath grasping spines—and their overall similarity to *bona fide* protoconodonts that become abundant in overlying Cambrian strata is suggestive of a connection. Whether or not these Ediacaran protoconodont-like SCFs derive from chaetognaths, their presence implies that bilaterians with gnathiferan-/chaetognath-like spines were present by the latest Ediacaran (as might be expected given the early appearance of phosphatic/phosphatized protoconodonts and articulated chaetognath grasping apparatus in rocks only a few million years younger [10]). Under the Chaetognathifera and Cucullophora (*Amiskwia + Timorebestia* + Chaetognatha as sister to the Gnathifera) hypothesis [20] where grasping spines are acquired in the chaetognath stem (figure 1*b*), this would mean the plesion leading to *Amiskwia*-like stem-chaetognaths had diverged from other stem-chaetognaths by the late Ediacaran occurrence of these spines (thus implying the Gnathifera had also already diverged from the branch leading to Chaetognatha by the late Ediacaran). An alternative possibility is that stem-Chaetognathifera possessed an equivalent of both grasping spines and gnathiferan-like internal jaws, but that *Amiskwia + Timorebestia* together derive from a chaetognath stem-group which lost the grasping spines. If instead chaetognaths reside within the Gnathifera, then this again could imply that stem-gnathiferans (or deeper bilaterian clades) possessed grasping spine-like structures—retained by modern chaetognaths, but lost in other gnathiferan clades. Under either scenario, semi-articulated arrays of Fortunian-age protoconodonts [10] indicate that the distinctive chaetognath-like grasping apparatus was an early innovation. Based on the position of chaetognaths within the Bilateria (as protostome lophotrochozoans (Spiralia) that are either within or sister to the Gnathifera [13,18,19]), fossil protoconodonts therefore constrain the divergence of the crown-Bilateria, Protostomia, Lophotrochozoa and depending on a more restrictive distribution of grasping spines, the divergence of Gnathifera and Chaetognatha all to the latest Ediacaran.

In addition to grasping spines, extant chaetognaths bear one or two rows of smaller ‘teeth’ that are differentiated from the larger, scimitar-shaped grasping spines [83]. Though their precise function is not well understood, these ‘teeth’ appear to be involved in injecting the prey (often copepods) with venom [119]. Despite the seeming abundance of grasping spines in Cambrian strata, fossil remains of disarticulated chaetognath ‘teeth’ are notably scarce. A few semi-articulated clusters of teeth have been reported adhered to larger grasping spines among phosphatic SSFs from the late Cambrian of Poland (fig. 7H–K of [9]). Teeth also appear to be rare as SCFs, though a cluster of ‘conical *Ceratophyton*-like elements’ among SCFs from the Paseky Shale (Cambrian Stage 4) of the Czech Republic (fig. 2M of [113]) may represent chaetognath teeth (figure 7). Articulated Cambrian chaetognath specimens that display more apomorphic features such as *Protosagitta spinosa* [12,22,23] and *Eognathacantha ercainella* [24] appear to possess teeth, but other articulated Cambrian chaetognaths show no evidence of differentiated teeth ([23,25]; it is unclear if an undescribed ‘crownward’ fossil chaetognath from Sirius Passet possesses teeth, fig. 5C of [21]). Scarcity of teeth among Cambrian protoconodonts could reflect a size/recognition bias, but may also indicate that many stem-chaetognaths (or chaetognath-like gnathiferans) lacked such teeth (fig. 8 of [26] provides a schematic hypothesis for how ‘teeth’ could have evolved from an apparatus of multi-bundled grasping spines).

The Cambrian fossil record also has implications for body-size evolution in the Chaetognatha and Gnathifera. Most extant chaetognaths are marine zooplankton predators, typically reaching lengths of a few millimetres (though a few exceed 10 cm and some appear to be secondarily benthic, e.g. spadellid chaetognaths [120]). The closely related gnathiferans are all minute, either comprising other zooplankton or meiofauna that occupy interstitial habitats [121]. In contrast, some Cambrian taxa were substantially larger. For instance, complete specimens of the Burgess Shale taxon *Capinatator praetermissus* reportedly reach up to 10 cm in length [25]. The Chengjiang chaetognath *Ankalodous sericus* is known only from the cephalic region, but extrapolation would also suggest a relatively large size for this taxon, as indicated by its grasping spines which reach 7 mm. Specimens of the chaetognath-like *Amiskwia* from the Burgess Shale range between 7.4 and 31.3 mm length (excluding tentacles) [30], but fossils of the comparable Sirius Passet taxon *Timorebestia koprii* reach up to 30 cm length [21]. Given the large sizes of some Cambrian chaetognaths, it is plausible, therefore, that Chaetognathifera evolved from ancestrally macroscopic benthic/nektobenthic worms [122], and that the Gnathifera are secondarily miniaturized. The chaetognath lineage appears to have taken to a pelagic (or at least nektobenthic) lifestyle relatively early, based on the widespread distribution of Cambrian protoconodonts, the presence of fin rays seemingly adapted for a nektonic habit in some Cambrian taxa, and the fossilized gut contents of some *Timorebestia* which include carapaces of the nektonic bivalved arthropod *Isoxys* [21].

## 5. Conclusions

The early fossil record of chaetognaths is crucial to resolving the evolutionary origins of the Chaetognatha and Gnathifera. Current records remain patchy, particularly among articulated macrofossils, yet some of the temporal signal can be assessed using disarticulated protoconodonts (at the expense of information regarding the broader morphology). New SCF material (this study) alongside a compilation of scattered SCF reports shows that organically preserved protoconodonts are a relatively abundant constituent of Cambrian organic microfossil preparations. Examination of this new record indicates that protoconodonts with a microstructure similar to chaetognath grasping spines occur in strata from the very earliest Cambrian, or even the late Ediacaran (figure 7). Importantly, even if these earliest spines are not derived from crown-chaetognaths, their presence still implies that bilaterians with chaetognath-like grasping spines had diverged from other deep bilaterian clades by the latest Ediacaran. By the time of the deposition of the major Burgess Shale-type Lagerstätten some approximately 15−20 million years later (Cambrian stages 3 through to Drumian stage), definitively chaetognath-like forms had evolved, with some fossils (e.g. *Protosagitta spinosa*) more closely resembling crown-group Chaetognatha (figure 7). Given the especially early appearance of protoconodont SCFs when compared with most other recognizable crown-bilaterians, continued exploration of this emerging dataset may also help to constrain the divergence of major bilaterian clades such as the Protostomia and crown-Bilateria more generally.

## Data Availability

Supplementary material is available online [123].
